# Coenzyme Q10 alleviates silicotic fibrosis in rats through the TGF-β1/Smad pathway

**DOI:** 10.3389/fphar.2026.1821205

**Published:** 2026-04-15

**Authors:** Jiping Yi, Yougen Wang, Hualing Zhang, Xiuhong Yang, Tianci Xiong, Huanhuan Xie, Lichao Zhan, Ming Zeng

**Affiliations:** 1 Hunan Prevention and Treatment Institute for Occupational Diseases, Affiliated Prevention and Treatment Institute for Occupational Diseases of University of South China, Changsha, China; 2 Nanchang Railway Disease Control and Prevention Institute, China Railway Nanchang Group Co., Ltd., Nanchang, China; 3 Department of Health Toxicology, Xiangya School of Public Health, Central South University, Changsha, China

**Keywords:** coenzyme Q10, rats, silicotic fibrosis, SiO_2_ dust, TGF-β1/Smad signaling pathway

## Abstract

**Background:**

Silicosis, the most prevalent occupational disease worldwide, currently lacks effective clinical treatments. Therefore, identifying an effective intervention and preventive drug is crucial for inhibiting silicosis fibrosis. Our preliminary experiments suggested that coenzyme Q10 (CoQ10) may alleviate fibrosis in a rat silicosis model, but the underlying mechanism remains unclear.

**Methods:**

In this study, a rat silicosis model was established via a single tracheal instillation of SiO_2_ dust suspension. Rats were then treated with CoQ10 at doses of 20, 50, and 125 mg/kg/d to investigate its mechanism in improving silica-induced inflammation and fibrosis.

**Results:**

Results showed that CoQ10 significantly reduced levels of inflammatory cytokines IL-1β and TNF-α in bronchoalveolar lavage fluid (BALF), alleviated lung tissue inflammation, oxidative stress, and collagen deposition. In the medium- and high-dose CoQ10 groups, expression of fibrosis-related proteins (α-SMA, Vimentin, Col-I, Col-III) decreased, while E-cad expression increased. Moreover, expression of TGF-β1, Smad2, and Smad3 was downregulated, and Smad7 expression was upregulated.

**Conclusion:**

These findings suggest that the anti-fibrotic effect of CoQ10 on silicosis may be associated with reducing pulmonary inflammation and oxidative stress, thereby inhibiting the TGF-β1/Smad signaling pathway. This study provides new insights into understanding the pathogenesis and potential treatment of silicosis.

## Graphical Abstract

**Figure:**
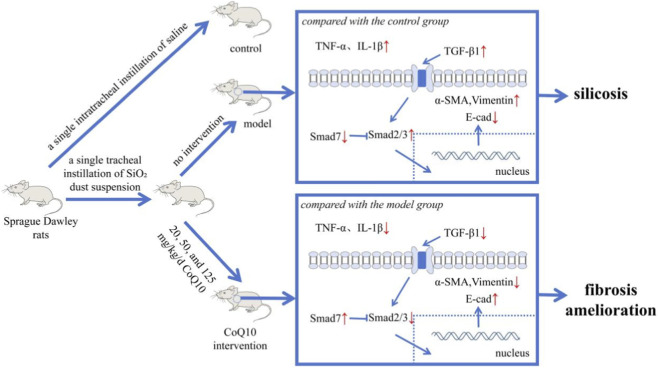

## 1 Introduction

Silicosis is an occupational disease characterized by acute and chronic inflammation with diffuse pulmonary fibrosis caused by long-term inhalation of free silica dust (SiO_2_), and it remains the most prevalent occupational disease worldwide (Krefft et al., 2020). In 2016, the Global Burden of Disease Study estimated that silicosis accounts for approximately 10,400 deaths annually and results in around 210,000 years of healthy life lost (Jamshidi et al., 2025). Despite the WHO/ILO initiative “Global Program for the Elimination of Silicosis,” the epidemic trend of this disease has not been effectively controlled in many countries. Currently, silicosis continues to exhibit a high incidence in industries such as coal mining, metallurgy, and building materials manufacturing, leading to significant health losses and socioeconomic burdens. However, there are no specific therapeutic methods for silicosis in clinical practice. Current treatment strategies primarily focus on anti-inflammatory and anti-fibrotic symptomatic treatments, combined with supportive therapies such as oxygen therapy, mechanical ventilation, and lung lavage, yet the efficacy remains unsatisfactory (Li et al., 2023; Li T. et al., 2022). Therefore, it is imperative to explore novel anti-silicosis therapeutic agents that are economical, low-toxicity, and capable of delaying disease progression, and to elucidate their underlying molecular mechanisms.

After entering the deep respiratory tract, finer SiO_2_ dust particles are retained in the lungs, triggering persistent inflammation (Yang et al., 2019) and oxidative stress (Nardi et al., 2018), which constitute the core mechanisms driving the progression of silicotic fibrosis. Chronic inflammation promotes the release of inflammatory factors such as interleukin-1 beta (IL-1β) and tumor necrosis factor-alpha (TNF-α) by macrophages and other cells, activating transforming growth factor-beta 1 (TGF-β1) signaling (Tang Q. et al., 2022). This induces epithelial-mesenchymal transition (EMT) in epithelial cells and drives the differentiation of fibroblasts into myofibroblasts, leading to the formation of granulomatous structures and advancing the fibrotic pathological process. A study by Liu (2008) also demonstrated that TNF-α and IL-1β not only directly stimulate fibrosis but also enhance the synthesis of plasminogen activator inhibitor-1 (PAI-1), thereby inhibiting the degradation of the extracellular matrix (ECM). Furthermore, reactive oxygen species (ROS) released due to SiO_2_ dust-induced oxidative stress exacerbates lung tissue injury and remodeling, synergistically promoting fibrotic development. Simultaneously, oxidative stress can regulate cell growth, proliferation, and apoptosis by inducing the expression of specific genes, such as the pro-fibrotic factor transforming growth factor-β (TGF-β), thereby facilitating the onset of pulmonary fibrosis. Consequently, suppressing inflammatory responses and oxidative stress has emerged a critical focus in the development of anti-silicosis drugs.

TGF-β is a multifunctional dimeric polypeptide growth factor comprising three common subtypes: TGF-β1, TGF-β2, and TGF-β3. Among these, TGF-β1 is the most prevalent and extensively studied subtype, closely associated with fibrosis, immunosuppression, and wound healing processes. TGF-β1 activates Smad-dependent pathways by binding to TβR-II and TβR-I receptors, promoting phosphorylation of mothers against decapentaplegic homolog 2/3 (Smad2/3), which then forms a complex with Smad4 and translocates into the nucleus to regulate target gene transcription. The TGF-β1/Smad signaling pathway is a core mechanism in the progression of silicotic fibrosis. On one hand, this pathway induces EMT in alveolar epithelial cells. EMT, as a significant source of mesenchymal cells (such as fibroblasts and myofibroblasts), actively participates in tissue repair or pathological processes, particularly tissue fibrosis, tumor invasion, and metastasis (Zhang et al., 2025). On the other hand, it promotes fibroblast proliferation and differentiation into myofibroblasts, leading to excessive deposition of ECM (e.g., collagen, fibronectin, and alpha-Smooth muscle actin) and ultimately causing destruction of lung tissue structure and fibrosis. Studies have shown that inhibiting TGF-β1/Smad signaling can effectively alleviate EMT and fibrosis progression, making this pathway a critical therapeutic target for silicotic fibrosis (Deng et al., 2024).

Coenzyme Q10 (CoQ10), an endogenously synthesized natural antioxidant, effectively prevents oxidation of proteins, lipids, and DNA and inhibits ROS generation (Fikry et al., 2024). It has garnered significant attention due to its diverse physiological functions, including anti-aging (Mantle and Hargreaves, 2022), cardioprotection (Raizner and Quiñones, 2021), anti-diabetic effects (Gholami et al., 2018), antioxidant activity (Wu et al., 2020), anti-inflammatory properties, and anti-fibrotic potential. Research indicates that exogenous CoQ10, as an endogenous substance, exhibits remarkable anti-inflammatory and antioxidant capacities. Its mechanism of action involves scavenging free radicals, enhancing the activity of antioxidant enzymes such as superoxide dismutase (SOD), reducing levels of the lipid peroxidation product malondialdehyde (MDA), and inhibiting ROS generation to mitigate oxidative stress. Simultaneously, CoQ10 suppresses the expression of key inflammatory factors such as TNF-α and interleukin-6 (IL-6). CoQ10 has demonstrated inhibitory effects in various organ fibrosis models (e.g., heart, liver, kidney, and lung). In a rat model of doxorubicin-induced myocardial fibrosis, CoQ10 significantly ameliorated fibrosis progression by suppressing the expression of TGF-β1 and connective tissue growth factor (CTGF) (Tarry-Adkins et al., 2016). Another study found that CoQ10 attenuated methotrexate-induced pulmonary fibrosis by activating autophagy-related pathways (Mohamed et al., 2019). Thus, as a natural antioxidant, anti-inflammatory agent, and potential anti-fibrotic compound, CoQ10 offers new directions and hope for the treatment of various diseases and research on fibrosis-related conditions.

In summary, chronic inflammation and oxidative stress play important roles in the development of silicotic fibrosis. The overexpression of inflammatory factors and oxidative stress promotes disease progression, and the TGF-β1/Smad signaling pathway plays a significant role in fibrosis development. CoQ10 has demonstrated potential in mitigating inflammation and oxidative stress in other disease models. Therefore, this study aims to establish a silica dust-induced rat model of silicosis and, under conditions of CoQ10 intervention, investigate changes in inflammatory factors, oxidative stress, pulmonary fibrosis levels, and molecules related to the TGF-β1/Smad signaling pathway, thereby clarifying the mechanism by which CoQ10 ameliorates inflammation and fibrosis in silicosis.

## 2 Materials and methods

### 2.1 Materials

#### 2.1.1 Ethics statement

All animal procedures were approved by the Animal Ethics Committee of Central South University (Approval No.: XYGW-2023-134). We meticulously conducted animal experimentation in strict adherence to the Guidelines for the Care and Use of Laboratory Animals.

#### 2.1.2 Experimental animals

Fifty male healthy SPF-grade Sprague-Dawley (SD) rats, weighing (200.0 ± 1.0) g, were purchased from Tianqin Biotechnology Co., Ltd. in Changsha, Hunan, China (Production License No.: SCXK (Xiang) 2019-0014). The rats were routinely housed in a barrier environment (temperature 23 °C ± 2 °C, humidity 55% ± 5%, 12-h light/dark cycle) with free access to food and water.

#### 2.1.3 Reagents

Free silica dust (SiO_2_, with ≥80% of particles measuring 0.5–5 μm, Sigma-Aldrich, United States); Coenzyme Q10 (Macklin Biochemical Technology Co., Ltd., Shanghai, China); Kits for Interleukin-1β (IL-1β), Tumor Necrosis Factor-α (TNF-α), Superoxide Dismutase (SOD), and Malondialdehyde (MDA) (Nanjing Jiancheng Bioengineering Institute, China); Hematoxylin and Eosin (H&E) staining solution, Mayer’s Hematoxylin, Eosin Y solution, Masson’s Trichrome Stain Kit, Reactive Oxygen Species (ROS) Staining Kit (Wuhan Biqindu Biotechnology Co., Ltd., China); Tween 20, Rapid Wright-Giemsa Stain, Bicinchoninic Acid (BCA) Protein Assay Kit (Beijing Dingguo Changsheng Biotechnology Co., Ltd., China); Antibodies against α-Smooth Muscle Actin (α-SMA), E-cadherin (E-cad), Vimentin, Collagen Type III (Col-III), Smad2, Smad3 (Abclonal Technology Co., Ltd., Wuhan, China); Antibodies against Collagen Type I (Col-I), Smad7 (Wuhan Sanying Biotechnology Co., Ltd., China); Antibody against Transforming Growth Factor Beta 1 (TGF-β1) (Abcam, China); Horseradish Peroxidase (HRP)-conjugated Goat Anti-Rabbit antibody (Abclonal, China).

### 2.2 Methods

#### 2.2.1 Animal modeling and grouping

SiO_2_ powder was ground in an agate mortar for 2 h, and microscopic examination confirmed that over 95% of the particles had a diameter less than 5 µm. The powder was dried at high temperature to constant weight. A precise amount of SiO_2_ powder was weighed using an analytical balance and suspended in physiological saline to create a 50 mg/mL suspension. This suspension was sterilized by autoclaving at 121 °C for 20 min. Prior to use, penicillin and streptomycin were added (400,000–600,000 units of penicillin and 1 g of streptomycin per 100 mL of suspension). A single non-exposed intratracheal instillation of 1 mL of this suspension was performed to establish the silicosis rat model. The rats were randomly divided into the following groups (n = 10 per group): silicosis model group, low-dose CoQ10 intervention group, medium-dose CoQ10 intervention group, and high-dose CoQ10 intervention group. An additional 10 rats received an intratracheal instillation of 1 mL of physiological saline as the control group. Twenty-four hours post-modeling, animals were randomly assigned to groups and administered the test substances: the low-, medium-, and high-dose CoQ10 intervention groups received CoQ10 orally at doses of 20, 50, and 125 mg/kg body weight (bw), respectively, in a gavage volume of 5 mL/kg bw, once daily. The silicosis model group and the control group received corn oil orally for 27 consecutive days.

#### 2.2.2 Sample collection

On day 28 after the intratracheal instillation of SiO_2_, rats were anesthetized by intraperitoneal injection of 60 mg/kg of 2% sodium pentobarbital and euthanized via abdominal aortic exsanguination. Rats were fixed on a dissection board, and the following samples were collected:

1. Bronchoalveolar Lavage Fluid (BALF): After anesthesia, the right main bronchus was ligated, and a tracheal cannula was inserted. The left lung was lavaged sequentially with 3 mL, 3 mL, and 2 mL of sterile physiological saline. The collected BALF was centrifuged, and the supernatant was aliquoted and stored at −80 °C. The cell pellet was resuspended in PBS for cell counting.
2. Lung Tissue Samples: The right middle lobe was fixed in 4% paraformaldehyde for subsequent histological analysis. The remaining lung lobes (upper, lower, and accessory lobes) were thoroughly rinsed with pre-cooled PBS to remove surface blood, and residual liquid was blotted with sterile filter paper. The tissues were then rapidly frozen in liquid nitrogen and transferred to a −80 °C ultra-low temperature freezer for long-term storage, intended for subsequent Western blot analysis and biochemical assays.

#### 2.2.3 Histopathological examination of lung tissue

Fixed lung tissues were dehydrated, cleared, embedded in paraffin, and sectioned. Sections were stained with H&E and Masson’s trichrome according to the respective kit instructions. After staining, slides were mounted with neutral balsam and observed under an optical microscope for imaging. Quantitative analysis of Masson staining was performed using ImageJ software.

#### 2.2.4 Determination of hydroxyproline (HYP) content in lung tissue by alkaline hydrolysis

Approximately 30–100 mg of lung tissue was accurately weighed, and 1 mL of hydrolysate was added. The mixture was incubated in a 95 °C water bath for 20 min, cooled to room temperature, pH-adjusted, and diluted to 10 mL with distilled water. An aliquot of the dilution was treated with activated charcoal to remove impurities by vortexing and centrifugation; the supernatant was used as the test sample. Following the assay kit instructions strictly, blank, standard, and test tubes were set up. Double-distilled water, standard solution, or test sample was added to the respective tubes, followed by reagent 1, reagent 2, and reagent 3. Tubes were incubated in a 60 °C constant temperature water bath for 15 min for complete color development. After cooling and centrifugation, the absorbance (OD value) of the supernatant was measured at 550 nm wavelength. The HYP content in the samples was calculated based on the standard curve.

#### 2.2.5 Total and differential cell counts in BALF

The BALF cell pellet was resuspended in 500 μL of PBS and mixed thoroughly. The total cell count was determined using a hemocytometer. A 50 μL aliquot of the cell suspension was used to prepare smears, which were air-dried, stained with Wright-Giemsa stain for 5–8 min, gently rinsed with running water, air-dried, and examined under a microscope. Two hundred cells were randomly counted to determine the percentages of macrophages and lymphocytes.

#### 2.2.6 Measurement of IL-1β and TNF-α levels in BALF by ELISA

After allowing the ELISA kits to equilibrate at room temperature for 1 h, standard wells, sample wells, and blank wells were set up according to the manufacturer’s instructions. Subsequently, 50 μL of standard or test sample was added to the respective wells. After incubation and washing, 100 μL of enzyme-conjugated antibody was added to all wells except the blank wells, followed by incubation at 37 °C for 1 h. After five washes, 100 μL of substrate A/B mixture was added to each well, and the plate was incubated in the dark for 15 min for color development. The reaction was stopped by adding stop solution, and the absorbance was measured at 450 nm. Sample concentrations were calculated based on the standard curve.

#### 2.2.7 Biochemical assay of SOD and MDA levels in lung tissue

A 10% lung tissue homogenate was prepared, and its total protein concentration was determined using the BCA method. For the SOD assay, control, control blank, test, and test blank wells were set up according to the kit instructions. Corresponding reagents, such as distilled water, test homogenate supernatant, enzyme working solution, enzyme diluent, and substrate application solution, were added sequentially. After mixing, the plate was incubated at 37 °C for 20 min, cooled to room temperature, and the absorbance was measured at 450 nm. Results were expressed as SOD activity units per milligram of tissue protein (U/mg prot).

For the MDA assay, blank, standard, test, and control tubes were set up according to the kit instructions. The following steps were performed sequentially: the blank tube received 0.1 mL of absolute ethanol, the standard tube received 0.1 mL of 10 nmol/mL standard solution, and the test and control tubes each received 0.1 mL of test sample; then, all tubes received 0.1 mL of reagent 1. After vortex mixing, 3 mL of reagent 2 and 1 mL of reagent 3 were added to all tubes; the control tube additionally received 1 mL of 50% glacial acetic acid. All tubes were sealed and reacted in a 95 °C water bath for 40 min, then cooled to room temperature under running water. The absorbance was measured at 532 nm, and the MDA content was calculated.

#### 2.2.8 DHE staining for ROS expression in lung tissue

Frozen lung tissue sections were warmed to room temperature, and the tissue areas were circled using a hydrophobic barrier pen. A 10 μmol/L DHE working solution was applied within the circles. The slides were placed in a humidified chamber and incubated at 37 °C in the dark for 30 min. Subsequently, the slides were washed three times with PBS in the dark, 5 min each. DAPI staining solution was then applied for nuclear counterstaining, incubated in the dark for 10 min, followed by three additional washes with PBS in the dark. Residual liquid was removed, an anti-fade mounting medium was applied, and coverslips were placed. Images were immediately observed and captured using a fluorescence microscope.

#### 2.2.9 Immunohistochemical staining for α-SMA positive areas in rat lung tissue

Paraffin-embedded rat lung tissue sections were baked at 60 °C for 4 h, followed by deparaffinization and rehydration, ending with a PBS rinse. Sections were placed in citrate buffer, and antigen retrieval was performed using a high-temperature high-pressure method. After natural cooling to room temperature, sections were washed with PBS three times for 5 min each. Then, 5% goat serum blocking solution was applied and incubated at room temperature for 30 min. After removing the blocking solution, a 1:200 dilution of the primary antibody against α-SMA was applied directly, and the sections were incubated overnight at 4 °C. The next day, after warming to room temperature, sections were washed thoroughly with PBS three times for 5 min each. An HRP-conjugated goat anti-rabbit secondary antibody was applied and incubated at 37 °C for 20 min, followed by three PBS washes. DAB substrate was used for color development, and nuclei were counterstained with Hematoxylin. After counterstaining, sections were dehydrated through a graded ethanol series, cleared in xylene, and finally mounted with neutral resin mounting medium and coverslips, allowing them to dry naturally at room temperature.

#### 2.2.10 Western blot analysis of protein expression in lung tissue

Collected lung tissues were added to an appropriate amount of high-efficiency RIPA lysis buffer and mechanically homogenized on ice. The homogenate was centrifuged at 12,000 rpm for 15 min at 4 °C, and the supernatant was collected for subsequent use. The protein concentration in the lung tissue lysates was determined using the BCA method. Thirty micrograms of total protein from each group were separated by SDS-PAGE and subsequently transferred to a PVDF membrane using the wet transfer method. The membrane was blocked with 5% non-fat milk for 2 h. After transfer, the membrane was cut into strips according to the pre-stained protein molecular weight markers. The strips were incubated with their respective primary antibodies (anti-GAPDH, 1:50,000; anti-E-cad, 1:2,000; anti-Vimentin, 1:20,000; anti-α-SMA, 1:5,000; anti-Collagen-I, 1:2,000; anti-Collagen-III, 1:2,000; anti-TGF-β1, 1:1,000; anti-Smad2, 1:1,000; anti-Smad3, 1:20,000; anti-Smad7, 1:1,000) overnight at 4 °C. The next day, the membranes were washed three times with TBST for 10 min each, incubated with an HRP-conjugated secondary antibody (1:5,000) at room temperature for 1 h, and then thoroughly washed again with TBST. An ECL chemiluminescent substrate was applied to the membranes, and signal images were captured using a chemiluminescence imaging system. Finally, the grayscale values of the bands were analyzed using ImageJ software. GAPDH was used as the internal reference to calculate the relative expression levels of the target proteins.

#### 2.2.11 Statistical analysis

All data were analyzed using SPSS 26.0 statistical software. Comparisons between two groups were performed using the T-test. For comparisons among multiple groups, one-way analysis of variance (ANOVA) was used for data meeting assumptions of normality and homogeneity of variances, while the rank-sum test was used for data that were non-normally distributed or had heterogeneous variances. A P-value of less than 0.05 was considered statistically significant.

## 3 Results

### 3.1 Silicotic fibrosis model in rats established by SiO_2_ dust suspension exposure

**FIGURE 1:**
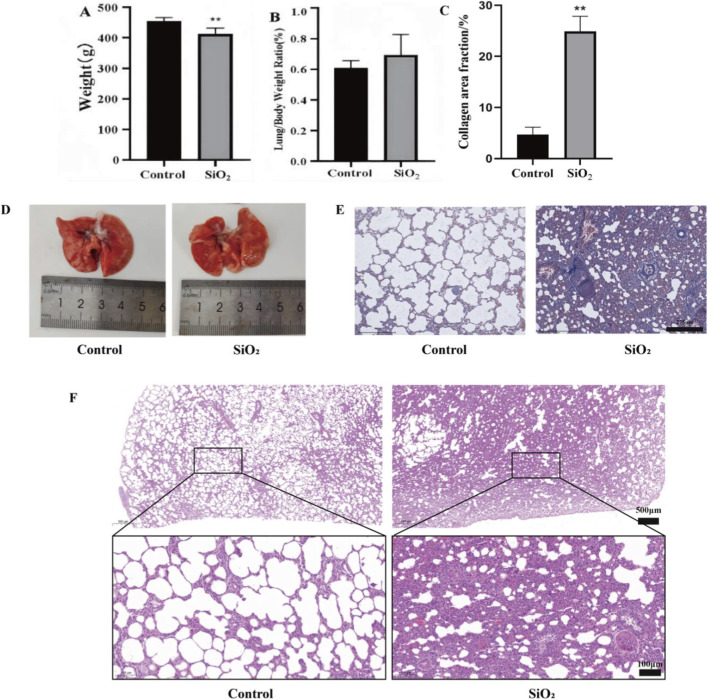
Establishment of a silicotic fibrosis rat model by SiO_2_ dust suspension exposure **(A)** Effect of SiO_2_ dust exposure on rat body weight; **(B)** Effect of SiO_2_ dust exposure on rat lung coefficient; **(C)** Collagen area fraction; **(D)** Gross morphological changes of rat lungs; **(E)** Masson staining (20×); **(F)** HE staining (10×, 40×). Note: **indicates P < 0.01 compared with the Control group. All data are presented as mean ± standard deviation (M ± SD). In Figure **(A,B)**, n = 10; in Figure **(C)**, n = 3.

After 28 days of feeding, the body weight of the model group was significantly lower than that of the control group (P < 0.01) (Figure 1A). The lung coefficient of the silicosis model group showed an increasing trend compared to the control group, but the difference was not statistically significant (Figure 1B). Gross morphological observation of the lungs revealed abnormally increased lung volume in the silicosis model group, with a noticeably hardened texture and significantly reduced elasticity. The lung surfaces were densely covered with white spherical protrusions, and some areas appeared grayish-white or even gray-black due to fibrosis and silica deposition (Figure 1D).

HE staining showed no significant morphological changes in the lung tissues of the control group, though minor inflammatory cell aggregation was observed in some areas. In contrast, the silicosis model group exhibited markedly thickened alveolar septa, extensive destruction of alveolar structures, alveolar collapse and atrophy, significant inflammatory cell infiltration, and the formation of nodular lesions in the lung tissues (Figure 1F). Masson’s staining indicated a significant increase in blue-stained areas in the lung tissues of the silicosis model group, with extensive diffuse fibrous tissue in the alveolar walls and widespread distribution of blue-stained collagen fibers in the vascular adventitia, bronchioles, and fibrotic consolidation areas (P < 0.01) (Figures 1C,E).

### 3.2 Intervention effect of CoQ10 on SiO_2_-induced pulmonary fibrosis in rats

**FIGURE 2:**
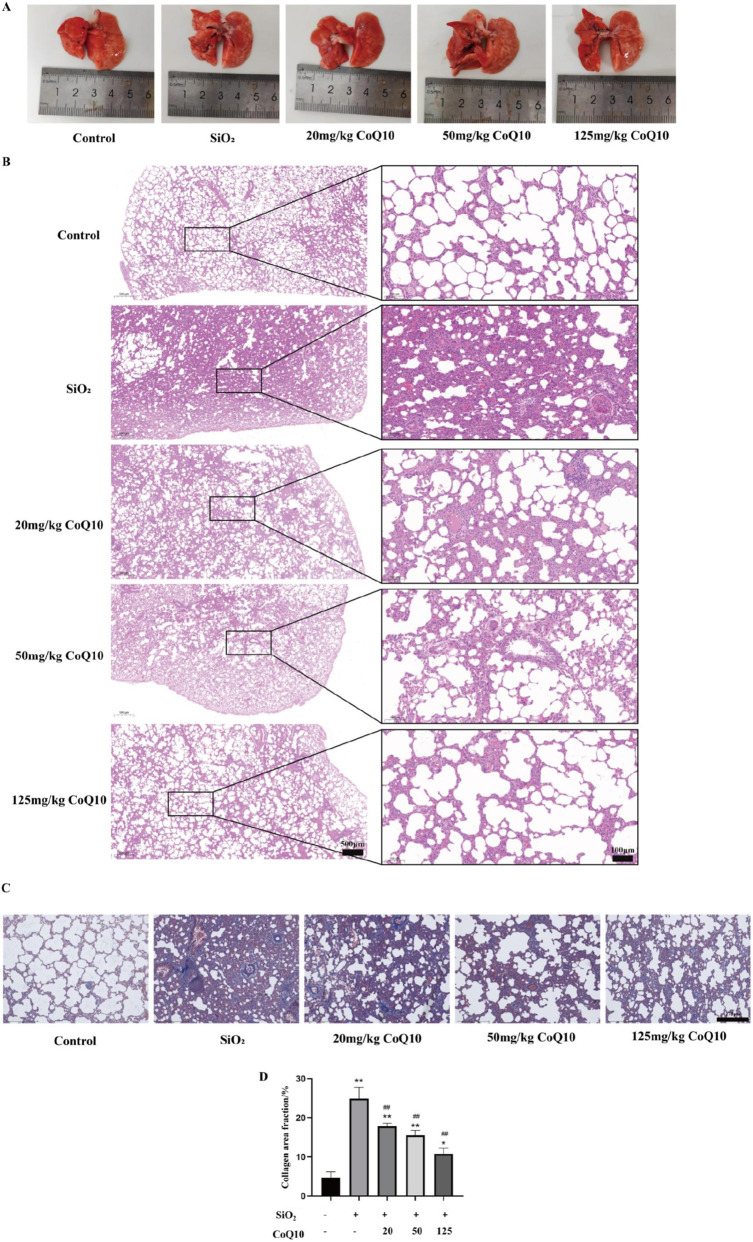
Effect of CoQ10 on pathological morphological changes in rat lungs induced by SiO_2_ dust suspension. (**(A)** Effect of CoQ10 on gross morphological alterations in rat lungs induced by silica dust; **(B)** HE staining of lung tissue (10×, 40×); **(C)** Masson staining of lung tissue (20×); **(D)** Collagen area fraction. ** indicates P < 0.01 compared with the Control group; ## indicates P < 0.01 compared with the Silicosis Model group. All data are presented as mean ± standard deviation (M ± SD), n = 3).

Gross anatomical observation revealed that, compared with the silicosis model group, the number of white spherical nodules on the lung tissue was significantly reduced in the low-dose CoQ10 intervention group. In the medium- and high-dose groups, these white spherical nodules were minimal and barely detectable (Figure 2A).

HE staining demonstrated that CoQ10 intervention ameliorated silica-induced lung injury in a dose-dependent manner. Compared with the silicosis model group, the medium- and high-dose CoQ10 intervention groups exhibited only slight thickening of the alveolar septa, along with reduced inflammatory cell infiltration and alveolar damage (Figure 2B). Masson staining indicated that CoQ10 intervention suppressed the extent of pulmonary fibrosis. Compared with the silicosis model group, the CoQ10-treated rats showed a reduction in both the distribution of collagen deposition and the area of blue staining (P < 0.01) (Figures 2C,D).

**FIGURE 3:**
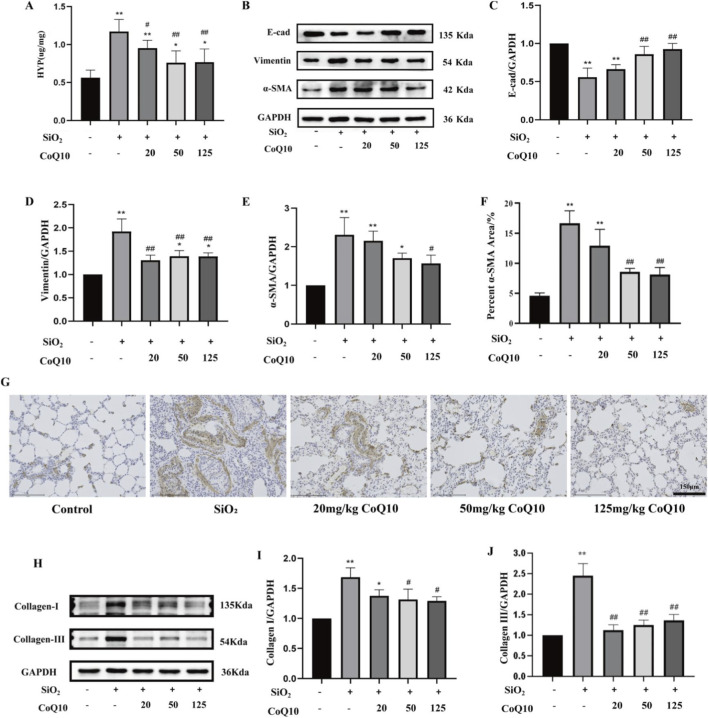
Effects of CoQ10 on fibrosis-related proteins in rat lung tissue induced by SiO_2_ dust suspension. (**(A)** Effect of CoQ10 on HYP content in rat lungs induced by silica dust; **(B)** Effect of CoQ10 on the expression of EMT-related proteins in rat lungs induced by silica dust; **(C)** E-cad protein expression level; **(D)** Vimentin protein expression level; **(E)** α-SMA protein expression level; **(F)** Immunohistochemical detection of α-SMA protein expression (10×); **(G)** α-SMA protein positive expression level; **(H)** Effect of CoQ10 on collagen protein expression in rat lung tissue induced by silica dust; **(I)** Collagen-I protein expression level; **(J)** Collagen-III protein expression level. Note: * indicates P < 0.05 compared with the Control group; ** indicates P < 0.01 compared with the Control group; # indicates P < 0.05 compared with the Silicosis Model group; ## indicates P < 0.01 compared with the Silicosis Model group. All data are presented as mean ± standard deviation (M ± SD), n = 3).

HYP is the main component of collagen, and its content in lung tissue can serve as an indicator for evaluating pulmonary fibrosis. Compared with the control group, the HYP content in the lung tissue of the silicosis model group was significantly increased (P < 0.01). In contrast, the HYP content in the CoQ10 intervention groups (low, medium, and high doses) was significantly reduced compared to the silicosis model group (P < 0.05 or P < 0.01) (Figure 3A).

WB results showed that, compared with the silicosis model group, CoQ10 intervention increased the protein level of E-cadherin in the medium- and high-dose groups (P < 0.01), decreased the protein level of Vimentin in all intervention groups (low, medium, and high doses) (P < 0.01), and significantly reduced the protein level of alpha-smooth muscle actin (α-SMA) in the high-dose group (P < 0.05) (Figures 3B–E). Immunohistochemistry results revealed that, compared with the control group, the expression of α-SMA around small bronchioles in the lung tissue of the silicosis model group was more extensive, with notable expression also observed in the alveolar wall septa (P < 0.01). However, after CoQ10 intervention, the expression range of α-SMA around small bronchioles in the medium- and high-dose groups was reduced compared to the silicosis model group, and no significant expression was detected in the alveolar wall septa (P < 0.01) (Figures 3F,G). This indicates that CoQ10 effectively inhibits the silica dust-induced EMT process.

WB results further demonstrated that the protein expression levels of Col-I and Col-III in the lung tissue of the silicosis model group were significantly elevated (P < 0.01). After CoQ10 intervention, Col-I expression was significantly reduced in the medium- and high-dose groups (P < 0.05), while Col-III expression was significantly suppressed in all dose groups (P < 0.01), suggesting that CoQ10 effectively mitigates the abnormal accumulation of fibrotic collagens (Figures 3H–J).

### 3.3 Effects of CoQ10 on oxidative stress levels and inflammatory response in rat lungs induced by silica dust

**FIGURE 4:**
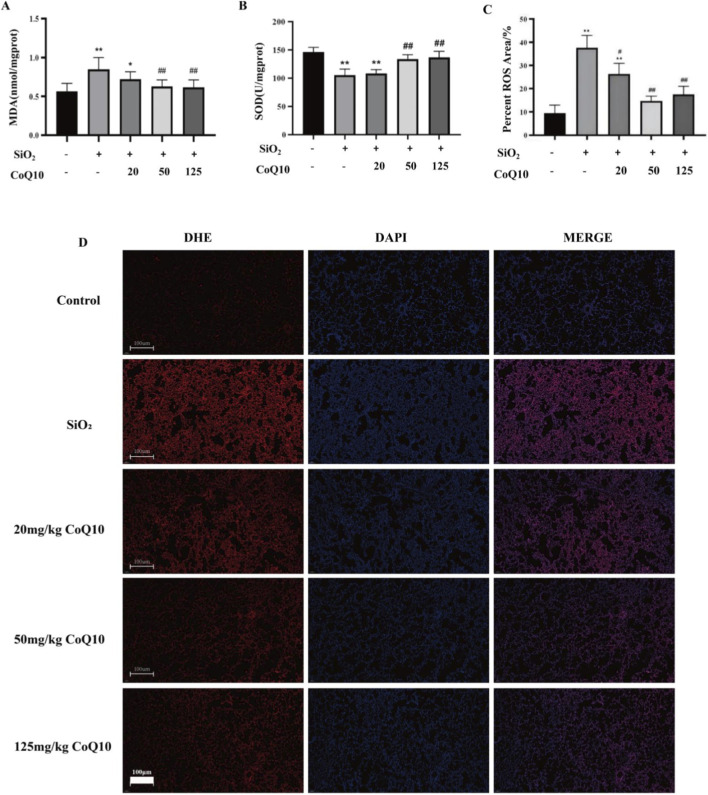
Effects of CoQ10 on oxidative stress levels in rat lungs induced by silica dust (**(A)** MDA content in rat lungs; **(B)** SOD activity in rat lungs; **(C)** Changes in ROS expression levels in rat lungs; **(D)** ROS expression in rat lungs detected by DHE staining (10×). Note: * indicates P < 0.05 compared with the Control group; ** indicates P < 0.01 compared with the Control group; # indicates P < 0.05 compared with the Silicosis Model group; ## indicates P < 0.01 compared with the Silicosis Model group. All data are presented as mean ± standard deviation (M ± SD), n = 3).

Compared with the silicosis model group, the medium- and high-dose CoQ10 intervention groups showed a decrease in MDA content and an increase in SOD activity in lung tissue (P < 0.01) (Figures 4A,B). DHE staining results revealed that the fluorescence intensity of ROS in the lung tissue of the silicosis model group was increased, covering almost the entire lung tissue. After CoQ10 intervention, the fluorescence intensity in the low-dose intervention group was reduced, with a smaller range of red signals, and the expression level of ROS was decreased to some extent compared to the silicosis model group (P < 0.05). Moreover, the fluorescence intensity and signal range in the medium- and high-dose CoQ10 intervention groups were significantly reduced (P < 0.01) (Figures 4C,D).

**FIGURE 5:**
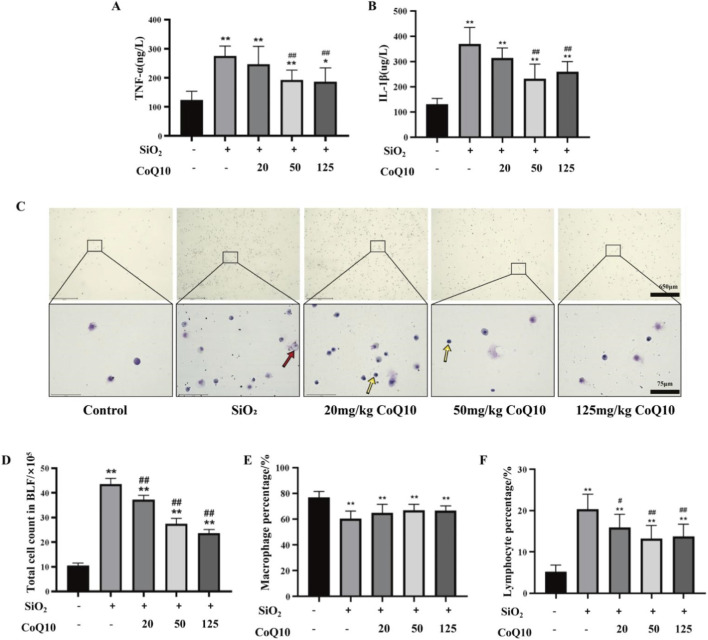
Effects of CoQ10 on the inflammatory response in rat lungs induced by silica dust (**(A)** TNF-α expression level in BALF; **(B)** IL-1β expression level in BALF; **(C)** Wright-Giemsa staining of cells in rat BALF (top: 4×, bottom: 40×); Red arrows indicate macrophages, and yellow arrows indicate lymphocytes. **(D)** Total cell count in BALF; **(E)** Macrophage percentage in rat BALF; **(F)** Lymphocyte percentage in rat BALF. Note: * indicates P < 0.05 compared with the Control group; ** indicates P < 0.01 compared with the Control group; # indicates P < 0.05 compared with the Silicosis Model group; ## indicates P < 0.01 compared with the Silicosis Model group. All data are presented as mean ± standard deviation (M ± SD), n = 3).

The levels of TNF-α and IL-1β in the BALF supernatant of the silicosis model group were significantly elevated (P < 0.01). Compared with the silicosis model group, the medium- and high-dose CoQ10 intervention groups showed a significant reduction in TNF-α and IL-1β levels (P < 0.01) (Figures 5A,B).

Wright-Giemsa staining was performed on BALF cell smears from each group, and the proportions of lymphocytes and macrophages among 200 counted cells were determined (Figure 5C). In BALF, macrophages are large (15–50 μm) with abundant cytoplasm containing vacuoles or phagocytic granules, and their nuclei are round, kidney-shaped, or horseshoe-shaped with fine chromatin. Lymphocytes are small (6–12 μm) with scant pale cytoplasm and a large, round, densely stained nucleus. The two cell types can be distinguished by these distinct morphological features. The results showed that the total cell count and lymphocyte percentage in the BALF of the silicosis model group were significantly increased, while the macrophage percentage decreased (P < 0.01). CoQ10 intervention effectively suppressed the silica dust-induced increase in total cell count and lymphocyte percentage in the BALF, but it did not significantly improve the reduced macrophage percentage (Figures 5D–F).

### 3.4 CoQ10 inhibits silica dust-induced activation of the TGF-β1/Smad signaling pathway in rat lungs

**FIGURE 6:**
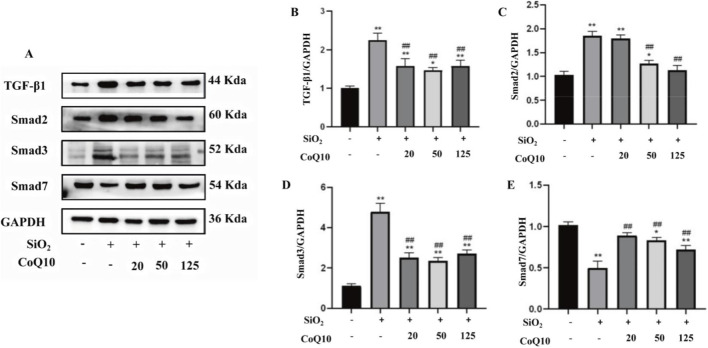
Effects of CoQ10 on the TGF-β1/Smad signaling pathway in rat lungs induced by silica dust (**(A)** Western blot analysis of TGF-β1/Smad pathway protein expression in rat lungs; **(B)** TGF-β1 protein expression level; **(C)** Smad2 protein expression level; **(D)** Smad3 protein expression level; **(E)** Smad7 protein expression level. Note: *P < 0.05 vs. Control group; **P < 0.01 vs. Control group; #P < 0.05 vs. Silicosis Model group; ##P < 0.01 vs. Silicosis Model group. All data are presented as mean ± standard deviation (M ± SD), n = 3).

Compared with the control group, the protein expression of TGF-β1, Smad2, and Smad3 in the lung tissue of the model group was significantly increased (P < 0.01), while the expression of Smad7 was significantly decreased (P < 0.01), indicating that silica dust exposure activated the TGF-β1/Smad signaling pathway in the lung tissue. In comparison to the silicosis model group, the expression of TGF-β1 and Smad3 proteins was significantly reduced in the lung tissues of the low-, medium-, and high-dose CoQ10 intervention groups (P < 0.01). However, the decrease in Smad2 protein expression was only statistically significant in the medium- and high-dose CoQ10 intervention groups (P < 0.01). In contrast, the expression of the negative regulator Smad7 was significantly elevated in all CoQ10 intervention groups (P < 0.01). These results suggest that CoQ10 intervention suppresses the activation of the TGF-β1/Smad signaling pathway induced by silica dust in rat lung tissue (Figures 6A–E).

## 4 Discussion

### 4.1 Establishment of the rat silicosis model

Currently, research related to the process of silica-induced pulmonary fibrosis can generally be divided into *in vivo* and *in vitro* approaches (Lam et al., 2022). For *in vitro* studies, researchers often use various cell types involved in the progression of silicosis, such as macrophages responsible for recognizing and phagocytosing SiO_2_ particles (Song et al., 2022), alveolar epithelial cells involved in EMT (Zhou et al., 2024), and fibroblasts and myofibroblasts responsible for substantial collagen deposition (Li et al., 2020; Ou et al., 2023). The advantage of these *in vitro* studies is the reduced use of experimental animals and the ability to microscopically dissect the pathological molecular mechanisms of the disease at the cellular level; hence, they are commonly used to identify signaling pathways involved in the initiation and progression of silicosis. For *in vivo* experiments, various animals can be used for silicosis modeling, with rodents, such as rats and mice, being the most frequently used. The advantage of *in vivo* studies lies in the ability to systematically observe the systemic responses following SiO_2_ exposure, including activation of the pulmonary immune system, oxidative stress, dynamic intermediate changes, and the fibrotic process – complex pathological processes that are difficult to fully replicate *in vitro* (Li R. et al., 2022). Furthermore, animal models allow for the observation of histological changes, such as alveolar structure collapse, inflammatory cell activation and recruitment, and collagen deposition, and enable the changes in key pathological indicators through molecular biology techniques. Therefore, *in vivo* studies hold an irreplaceable role in both basic research on silicosis and the exploration of treatments.

In the present study, we established the model by performing a single intratracheal instillation of 1 mL of a 50 mg/mL SiO_2_ dust suspension in SD rats anesthetized with isoflurane. This modeling approach has been repeatedly demonstrated in our laboratory’s previous studies to successfully establish a rat silicosis model (Tang M. et al., 2022), hence we continued its use.

### 4.2 CoQ10 alleviates silicosis fibrosis in rats

In previous studies, CoQ10 has primarily been used clinically for the treatment of cardiovascular diseases (Arenas-Jal et al., 2020). Recent studies have also found that CoQ10 can alleviate fibrosis in multiple organs such as the heart, liver, and intestines (Alhusaini et al., 2023; Hargreaves and Mantle, 2019; Mohamed and Said, 2021).These findings clearly suggest that CoQ10 may also play an important role in pulmonary fibrotic diseases. In this study, rats were intervened with three doses of CoQ10 (20 mg/kg, 50 mg/kg, and 125 mg/kg; the highest dose was selected based on the human no-observed-adverse-effect level of 1,200 mg/d, considering an adult weight of 60 kg and a human-to-rat conversion factor of 6-7) for 27 days. The study found that the lungs of the silicosis model group appeared grayish-white grossly, with most rats exhibiting numerous white spherical nodules on the lung surface. Furthermore, possibly due to the presence of fibrous tissue leading to compensatory hyperplasia, the lung volume in the model group was larger than that in the CoQ10 intervention groups, consistent with previous findings (Komai et al., 2019). In contrast, rats treated with 50 mg/kg and 125 mg/kg CoQ10 had very few, macroscopically undetectable white spherical nodules on their lungs, and the lung volume was generally smaller compared to the silicosis model group. Pathologically, H&E staining of the model group lungs showed significant collapse of lung structure, thickened alveolar walls, inflammatory cell infiltration, and the formation of fibrotic nodules. Conversely, intervention with medium and high doses of CoQ10 resulted in reduced areas of alveolar structure collapse, absence of obvious fibrotic nodules, and decreased inflammatory cell infiltration in the rat lungs. Evidently, CoQ10 alleviated the SiO_2_ particle-induced structural alterations in the rat lungs.

Concurrently, SiO_2_ particles entering the lungs can also lead to severe diffuse pulmonary fibrosis (Tian et al., 2023). The Masson staining results in this experiment revealed that the extent of collagen deposition in the lungs of CoQ10-treated rats was significantly reduced compared to the silicosis model group, and no obvious focal fibrous tissue hyperplasia, as seen in the model group, was observed between the alveolar walls. Hydroxyproline (HYP), a major component of collagen, is also a key indicator in fibrosis research (Wang Y. et al., 2023). The results of this experiment showed that the HYP content in the lung tissues of CoQ10-treated rats was significantly reduced to some extent compared to the silicosis model group. Research indicates that after SiO_2_ particles enter the alveoli, they continuously irritate the alveolar spaces and induce EMT in alveolar epithelial cells through the secretion of various inflammatory factors, thereby generating more myofibroblasts. Myofibroblasts are crucial cells driving pulmonary fibrosis; their abnormal proliferation leads to excessive synthesis and secretion of extracellular matrix components, such as collagen and elastin, directly triggering interstitial fibrosis (Wang X. C. et al., 2023). This study found that CoQ10 significantly increased the expression of the epithelial marker E-cadherin protein while decreasing the expression of EMT-related mesenchymal marker proteins α-SMA and Vimentin in lung tissue, indicating that CoQ10 alleviated the EMT process in alveolar epithelial cells. Regarding proteins directly associated with fibrosis, the expression of Collagen-I and Collagen-III proteins in the lung tissues of CoQ10-treated rats was also significantly lower than in the silicosis model group.

### 4.3 CoQ10 reduces silicosis fibrosis in rats via antioxidant and anti-inflammatory effects

Oxidative stress refers to an imbalance between ROS and the antioxidant defense system within the organism, triggering a series of adaptive responses. After SiO_2_ dust enters the human body, fractured crystalline silica generates a large number of siloxy radicals, leading to excessive formation of ROS and reactive nitrogen species (RNS). Additionally, inflammation induced by SiO_2_ particles also produces more ROS (Li et al., 2024; Zhu et al., 2022). If the redox homeostasis is disrupted, causing excessive ROS generation and insufficient clearance capacity, ROS will accumulate within cells, initiating an oxidative stress cascade reaction that ultimately induces lung tissue injury and promotes its abnormal remodeling. MDA, a well-known secondary product of lipid peroxidation, is often a useful biomarker for assessing oxidative stress levels (Tsikas, 2017). Studies by Sharawy et al. (2013) found that MDA levels in SD rats stimulated with SiO_2_ dust suspension were significantly higher than in the control group. SOD is a key antioxidant enzyme widely present in various cells. Its primary function is to catalyze the conversion of superoxide radicals (O_2_^−^) into hydrogen peroxide (H_2_O_2_) and oxygen, thereby reducing intracellular oxidative damage (Wang et al., 2018). Results from one study (Li et al., 2021) indicated that SOD activity in the lung tissue of the silicotic fibrosis model group was significantly lower compared to the control group, indicating a decline in the antioxidant capacity in the rat lungs. In the results of this study, rats treated with CoQ10 showed a significant reduction in ROS expression in lung tissue compared to the silicosis model group, and the content of MDA also decreased markedly. The reason may be that CoQ10, acting as an electron carrier between Complex I (NADH dehydrogenase), Complex II (succinate dehydrogenase), and Complex III (cytochrome c reductase), maintains the integrity of the intracellular mitochondrial electron transport chain (ETC), reducing the accumulation of O_2_^−^ due to electron leakage, thereby lowering mitochondrial oxidative stress levels (He et al., 2024). Simultaneously, the reduced form of CoQ10 (ubiquinol) can directly neutralize O_2_^−^, hydroxyl radicals (·OH), etc., blocking oxidative damage caused by free radicals to cell membranes, mitochondria, and the nucleus (Gutierrez-Mariscal et al., 2019). Furthermore, some researchers suggest that CoQ10 can further scavenge lipid radicals in the lipid peroxidation chain reaction by regenerating exogenous antioxidants like vitamin E, inhibiting the continuous oxidative destruction of membrane phospholipids (Gutierrez-Mariscal et al., 2019).

Besides oxidative stress, the inflammatory response also plays a critical role in the initiation and progression of pulmonary fibrosis. After SiO_2_ dust enters the lung tissue, pulmonary macrophages are rapidly activated, come into contact with and phagocytose SiO_2_ particles, leading to macrophage destruction and rupture, releasing a large number of inflammatory mediators, including key factors like IL-1β and TNF-α (Qin et al., 2021). In related studies, these inflammatory factors not only promote the occurrence of EMT but also exacerbate the local immune response, recruiting lymphocytes, neutrophils, and fibroblasts, thereby further enhancing lung tissue damage (Huang et al., 2023). Therefore, the inflammatory response is likely another crucial link in the development of silica dust-induced pulmonary fibrosis. In this study, pathological observation in this experiment revealed massive inflammatory cell infiltration in HE-stained lung tissue sections of the silicosis model group. However, after CoQ10 intervention, the areas of inflammatory cell infiltration were significantly reduced in all treatment groups. This study also detected inflammatory factors in the rat BALF supernatant and found that CoQ10 at doses of 50 mg/kg and 125 mg/kg significantly reduced the SiO_2_ suspension-induced expression of IL-1β and TNF-α in rat BALF. This is because CoQ10 can reduce the release of mitochondrial ROS and mtDNA, thereby inhibiting the activation of the NF-κB/NLRP3 inflammasome axis, ultimately reducing the secretion of pro-fibrotic factors like TGF-β, IL-1β, and TNF-α (Antar et al., 2024). After CoQ10 intervention, the total cell count in the BALF of rats in all dose groups was significantly reduced compared to the model group. Meanwhile, Wright-Giemsa staining of smears from the resuspended BALF cells showed a significant increase in the lymphocyte percentage and a decrease in the macrophage percentage in the silicosis model group. These results suggest that after SiO_2_ particles enter the rat lungs, macrophages phagocytose the retained SiO_2_ particles en masse, subsequently rupturing and leading to a decreased macrophage percentage. The ruptured macrophages release large amounts of inflammatory factors like IL-1β and TNF-α, recruiting more lymphocytes to the site of alveolar injury, resulting in an increased lymphocyte percentage, consistent with findings from studies by Sharawy, El-Kashef DH, et al. (El-Kashef, 2018; Sharawy et al., 2013). After CoQ10 intervention, all dose groups significantly reduced the lymphocyte percentage in rat BALF.

### 4.4 CoQ10 alleviates silicosis fibrosis in rats by suppressing the activation of the TGF-β1/Smad signaling pathway

The TGF-β subfamily itself includes three common isoforms: TGF-β1, TGF-β2, and TGF-β3 (Lodyga and Hinz, 2020). Among these, TGF-β1 is the most extensively studied. The TGF-β1/Smad signaling pathway it mediates can regulate target gene transcription and protein expression, thereby inducing fibrosis in various tissues and organs (Hao et al., 2019). Smad proteins are specific intracellular signal transduction molecules for the TGF-β family. TGF-β1 stimulation leads to phosphorylation of Smad2/3, which translocates to the nucleus and participates in EMT, fibroblast proliferation, and myofibroblast differentiation, ultimately promoting the occurrence of fibrosis. Additionally, Smad7 is a negative regulator within the TGF-β signaling pathway. Its normal expression can, to some extent, ameliorate tissue damage and antagonize the development of fibrosis (Yan et al., 2016). It is also noteworthy that inflammatory responses and excessive oxidative stress can activate the TGF-β1/Smad signaling pathway in tissues and organs. Recent research has found that CCl_4_ can activate the TGF-β1/Smad pathway in the liver, promoting hepatic fibrosis, by inhibiting the Nrf2 pathway and enhancing the NF-κB pathway (Wang Z. et al., 2023). Studies by Gorowiec et al. (Gorowiec et al., 2012) demonstrated that externally applied oxidative stress can enhance the expression and activity of TGF-β1, thereby inducing the transition of pulmonary epithelial cells to a mesenchymal phenotype. In another study, a pharmacological Nrf2 enhancer was shown to promote Smad7 expression by boosting antioxidant capacity, subsequently blocking Smad2/3 phosphorylation and inhibiting the TGF-β1-Smad2/3 signaling pathway, thereby alleviating renal fibrosis (Song et al., 2019). Clearly, the synergy between inflammation and oxidative stress can further activate the TGF-β1/Smad signaling pathway, induce EMT in epithelial cells, and stimulate collagen secretion by myofibroblasts, accelerating fibrosis progression. As discussed in previous sections, excessive inflammatory response and oxidative stress are typical manifestations in rat silicotic fibrosis.

In this study, the rat fibrosis model induced by SiO_2_ dust suspension showed significant activation and dysregulation of the TGF-β1/Smad signaling pathway. Compared to the control group, the expression of TGF-β1, Smad2, and Smad3 proteins was significantly increased in the lung tissue of the silicosis model group, while Smad7 expression decreased. This aligns with previous *in vivo* and *in vitro* findings in silicosis research (Feng et al., 2020; Zhao et al., 2024). Concurrently, CoQ10 intervention significantly reduced the expression of TGF-β1, Smad2, and Smad3 proteins and upregulated Smad7 protein expression in the lung tissues of the intervention groups. Therefore, the regulation of the TGF-β1/Smad signaling pathway is a core mechanism underlying CoQ10’s anti-fibrotic effect in rat silicosis. This regulation may be achieved through multiple aspects: On one hand, as shown in our results above, CoQ10 can reduce the expression of ROS and inflammatory factors such as IL-1β and TNF-α. These components have been proven in related studies to facilitate the conversion of latent TGF-β1 to its active form, thereby inducing abnormal initiation of Smad-dependent signaling (Xu et al., 2022). On the other hand, during the chronic inflammation phase, activated M2 macrophages and T lymphocytes can release large amounts of latent TGF-β1, and mechanisms involving integrins and MMPs promote its conversion to the biologically active form, exacerbating the fibrotic process (Annes et al., 2003). Our previous discussion indicated that CoQ10 can reduce the recruitment and activation of macrophages and lymphocytes, thereby indirectly decreasing the formation and release of active TGF-β1. Furthermore, studies have shown that activation of the nuclear factor Nrf2 can promote the expression of the negative regulatory protein Smad7, thus blocking Smad2/3 phosphorylation and their nuclear translocation, and delaying fibrosis progression (Shu et al., 2021). Some research has found that CoQ10 can activate the Nrf2 signaling pathway, suggesting it may exert anti-fibrotic effects indirectly by modulating the Nrf2/Smads axis (Tripathi et al., 2023). Evidently, these results confirm that CoQ10 has an inhibitory effect on the activation of the TGF-β1/Smad signaling pathway in the lungs of silica dust-exposed rats. Moreover, the anti-fibrotic effect of CoQ10 on rat silicosis is likely related to its ability to alleviate pulmonary inflammation and oxidative stress, thereby inhibiting the expression of the TGF-β1/Smad signaling pathway and mitigating the progression towards fibrosis in rat silicosis.

## 5 Conclusion

The anti-fibrotic effect of CoQ10 on silicosis in rats may be attributed to its ability to alleviate pulmonary inflammation and oxidative stress, thereby inhibiting the TGF-β1/Smad signaling pathway. This finding provides new insights for exploring the pathogenesis and treatment strategies of silicosis.

## Data Availability

The original contributions presented in the study are included in the article/supplementary material, further inquiries can be directed to the corresponding author.
